# Sleep and nighttime energy consumption in early childhood: a population‐based cohort study

**DOI:** 10.1111/ijpo.12006

**Published:** 2015-01-07

**Authors:** L. McDonald, J. Wardle, C. H. Llewellyn, L. Johnson, C. H. M. van Jaarsveld, H. Syrad, A. Fisher

**Affiliations:** ^1^ Health Behaviour Research CentreDepartment of Epidemiology and Public HealthUniversity College LondonLondonUK; ^2^ School for Policy StudiesUniversity of BristolBristolUK; ^3^ Department of Primary Care and Public Health SciencesKing's College LondonLondonUK

**Keywords:** diet, energy intake, sleep

## Abstract

**Background:**

Shorter sleep is a risk factor for weight gain in young children. Experimental studies show that sleep deprivation is associated with higher nighttime energy intake, but no studies have examined the patterning of energy intake in relation to nighttime sleep duration in young children.

**Objectives:**

The objectives of the study were to test the hypothesis that shorter‐sleeping children would show higher nighttime energy intake and to examine whether the additional calories were from drinks, snacks or meals.

**Methods:**

Participants were 1278 families from the Gemini twin cohort, using data from one child per family selected at random to avoid clustering effects. Nighttime sleep duration was measured at 16 months of age using the Brief Infant Sleep Questionnaire. Energy intake by time of day and eating episode (meal, snack, drink) were derived from 3‐day diet diaries completed when children were 21 months.

**Results:**

Consistent with our hypothesis, shorter‐sleeping children consumed more calories at night only (linear trend *P* < 0.001), with those sleeping <10 h consuming on average 120 calories (15.2% of daily intake) more at night than those sleeping ≥13 h. The majority of nighttime intake was from milk drinks. Associations remained after adjusting for age, sex, birth weight, gestational age, maternal education, weight and daytime sleep.

**Conclusions:**

Shorter‐sleeping, young children consume more calories, predominantly at night, and from milk drinks. Parents should be aware that providing milk drinks at night may contribute to excess intake. This provides a clear target for intervention that may help address associations between sleep and weight observed in later childhood.

## Introduction

Short sleep has been associated with obesity risk in children, adolescents and adults, but the association appears stronger at younger ages 1, 2. Within paediatric populations, short sleep has been shown to raise the risk of overweight and obesity by more than 60% 1, 2 and is associated with greater fat mass 3 and abdominal adiposity 4.

Both higher energy intake and lower energy expenditure are potential pathways linking shorter sleep to adiposity. However, sleep is an energy‐efficient activity and the fact that more hours of wakefulness promote weight gain particularly implicates higher energy intake as the underlying mechanism 5. Studies in which sleep duration is experimentally modified over short periods have consistently shown effects on food intake 6, 7, 8. For example, a recent intervention with 8–11‐year‐old children showed that decreasing sleep duration to 7 h a night for a week led to an 8% increase in energy intake and 0.24 kg of weight gain 9. Epidemiological data also find that shorter sleep in early life is associated with higher energy intake and this effect is observed before the association with weight has emerged 10.

Increased time available for consumption has been proposed as one of the mechanisms that could drive higher energy intake in shorter sleepers 5. Experimentally delaying bedtime in conditions of *ad libitum* access to food increased energy intake at night, particularly during the hours of extended wakefulness 6, 7. We have shown that short‐sleeping children tend to have a later bedtime rather than an earlier wake time 11, suggesting that they may be particularly susceptible to overconsumption in the evening. However, no studies have examined whether habitually shorter‐sleeping, young children consume more at night.

The aim of the present study was therefore to test the hypothesis that energy intake would be specifically higher in the evening in shorter‐sleeping children by examining the temporal patterning of energy intake in relation to sleep duration using 3‐day dietary data. The secondary aim was to determine whether the additional calories were from meals, snacks or drinks.

## Methods

### Participants

Participants were 1278 families participating in the Gemini study. Gemini is a representative population‐based cohort of twins set up to examine early life influences on growth 12. The baseline sample included 2402 families with twins born in England and Wales between March and December 2007 (36% of all live twin births). As common in cohort studies, lower socioeconomic groups were somewhat underrepresented, the majority (95%) were from a ‘white’ ethnic group and participating families were slightly healthier than average (lower parental smoking and body mass index [BMI]) 12. Data were from families who provided complete diet and sleep data (*n* = 1278, 53.2% baseline sample). Children with complete data had mothers who were slightly older (34.6 vs. 32.5 years), had slightly lower BMI at baseline (24.7 vs. 25.6), had slightly higher education levels and were more likely to be from a white ethnic background (*P's* all <0.001). Intraclass correlations between twins for the sleep and dietary data were high; therefore, to avoid clustering effects by family, one twin was randomly selected from each pair. Informed written consent was provided by all parents. Ethical approval was granted by the University College London Committee of Non‐National Health Service Human Research.

### Measures

#### Sleep

Sleep was assessed when the children were approximately 16 months old (mean 15.7, standard deviation [SD] 1.1 months) using an adapted version of the Brief Infant Sleep Questionnaire 13. Parents reported how long their child usually slept during the day and their bedtime and wake time. Nighttime sleep duration was calculated from bedtime and wake time. This method has been validated against actigraphy 13; and in a subsample of 40 Gemini families, 1 week test‐retest reliability was excellent (intraclass correlation 0.89; 95% confidence interval 0.76–0.95 for sleep duration). Given the uncertainty in the literature over how best to define ‘short’ sleep 14, we identified five sleep groups: <10 h a night, 10 to <11 h, 11 to <12 h, 12 to <13 h and ≥13 h a night, which allowed a test of linear associations. The same groupings had been used to examine daily energy intake in a previous study and showed a linear trend of increasing energy intake with decreasing sleep duration 10. Associations with 24‐h sleep were not examined because daytime sleep may have different functions from nighttime sleep and is not associated with obesity risk in prospective studies of children 15, but we controlled for daytime sleep in all analyses.

#### Dietary intake

Dietary data were collected when children were on average 21 months old (mean 20.7, SD 1.3 months). Parents were sent diet diaries with detailed instructions and asked to record everything their child ate or drank inside or outside the home for 3 d, including two weekdays and one weekend day while in their care. Where possible, parents were asked to provide household measures and packet weights to estimate food consumed. In the absence of this, parents were asked to provide portion size estimates using instructions from a photographic booklet 16. Each dietary day covered a 24‐h period and for each entry, parents recorded the time of the eating occasion.

All food and drink items within a single time entry constituted an eating occasion. Each occasion was coded as a meal or snack using a food‐based classification system 17. If an eating occasion included a ‘meal’ food item (e.g. meat, eggs, cooked vegetables), it was coded as a meal and if it consisted of one or more snack items (e.g. crisps, yogurt, raw vegetables) without a meal item, it was coded as a snack. A drink occasion was defined as a drink without any foods. This coding frame ensured meal, snack and drink occasions were consistently defined. Parents were asked to provide information on whether each occasion was a meal, snack or drink, but a large proportion of these data were missing. However, the agreement rate between the coding frame and parents' classification was high (κ = 0.82, *P* < 0.001).

Nutrition data were coded by researchers at the Medical Research Council Human Nutrition Research unit. Energy intakes were estimated by matching each food reported in the diary to a food in the National Diet and Nutrition Survey food composition database then multiplying it by portion size estimate from the information provided by the parent. Some general dietary questions were included with the diaries to assist in coding missing data. Dietary variables examined were total energy intake (mean daily kcal over 3 d) and energy intake from meal, snack and drink occasions (mean daily kcal). This method of dietary data collection outperforms alternative unweighed methods and has been validated against weighed dietary records in children aged 6 to 24 months 18.

#### Sociodemographic and weight data

Sex, birth weight, gestational age and maternal education were reported by the primary caregiver in baseline questionnaires. Maternal educational attainment was dichotomized to lower (no university level education; 51%) or higher (university education; 49%). Gemini families are asked to provide weight measures every 3 months; weight data close to sleep data were available for 84% of the sample.

### Statistical analyses

#### The temporal patterning of energy intake

Dietary and sleep data were normally distributed. To examine the temporal patterning of energy intake, the 24‐h dietary day was divided into four periods designed to cover the range of conventional meal/snack intakes for young children in the UK: morning (6:00 to <10:00 h), daytime (10:00 to <15:00 h), afternoon/evening (15:00 to <19:00 h) and night (19:00 to <6:00 h). The start of the nighttime period was fixed according to the average bedtime in the sample (19:12 h) and the end of this period was slightly before the average wake time of 6:50 in the morning. Univariate analysis of variance (anova) compared energy intake between the five sleep groups for each time period. Tests of a linear association (polynomial contrasts) were used to determine whether energy intake increased or decreased with sleep duration for any given time period.

#### Partitioning energy intake into meals, snacks and drinks

For any time period where shorter sleepers reported higher energy intake, univariate anova using tests of a linear association examined whether sleep was associated with differences in energy intake from meals, snacks or drinks.

All univariate analyses were repeated adjusting for age, sex, birth weight, gestational age, maternal education, weight and daytime sleep duration in analyses of covariance with polynomial contrasts (testing for a linear association). Daytime sleep is common in children of this age and was included as a confounder in case shorter nighttime sleepers were compensating with more sleep during the day. Data from boys and girls were combined as there were no significant interactions between energy intake, sleep and gender.

## Results

Participants' characteristics by nighttime sleep duration are presented in Table 1. There were no linear associations between sociodemographic variables or daytime sleep and nighttime sleep duration. Shorter sleepers had both a later bedtime and an earlier waketime. Nighttime sleep was not associated with weight at this age.

**Table 1:** Participant characteristics by nighttime sleep duration (mean [standard deviation] unless otherwise stated)

	<10 h	10–<11 h	11–<12 h	12–<3 h	≥13 h	*P* (linear trend)	*P* (between groups)
Total (*n* = 1278)	36	129	599	437	77		
Age at sleep record (months)	15.67 (1.10)	15.78 (1.08)	15.73 (1.07)	15.75 (1.17)	15.67 (0.96)	0.919	0.944
Age at diet diary record (months)	20.50 (0.94)	20.73 (0.98)	20.74 (1.14)	20.74 (1.25)	20.39 (0.98)	0.653	0.101
Gender (%)							
Boy (*n* = 619)	50.0	59.7^b^	52.8^b^	39.4	46.8		
Girl (*n* = 659)	50.0	40.3^b^	47.2^b^	60.6	53.2	0.194	<0.001
Birth weight (kg)	2.31 (0.51)	2.38 (0.55)	2.49 (0.53)	2.47 (0.52)	2.38 (0.53)	0.314	0.050
Gestational age (weeks)	35.92 (2.80)	36.05 (2.97)	36.29 (2.46)	36.30 (2.18)	35.87 (2.30)	0.874	0.445
Maternal education (%)							
Low (*n* = 499)	66.7	43.4	35.4^c^	38.2^c^	51.9		
High (*n* = 779)	33.3	56.6	64.6^c^	61.8^c^	48.1	0.086	<0.001
Daytime sleep (hours)	1.93 (0.86)	1.92 (0.76)	1.83 (0.61)	1.89 (0.69)	1.79 (0.80)	0.258	0.441
Bedtime	9:08 (1:14)	7:46 (0:51)	7:33 (0:30)	6:58 (0:31)	6:33 (0:34)	<0.001	<0.001
Wake time	6:15 (1:08)	6:12 (0:47)	6:38 (0:30)	7:11 (0:32)	7:49 (0:42)	<0.001	<0.001
Weight (kg)^a^	10.22 (1.20)	10.87 (1.67)	10.93 (1.58)	10.90 (1.50)	10.87 (1.50)	0.063	0.213

a Weight data available for 84% of the sample.

b Significantly different from 12‐ to 13‐h group.

c Significantly different from <10‐h group.

### The temporal patterning of energy intake

Dietary data by sleep duration are presented in Table 2. Energy intake in the morning (6:00 to <10:00 h) and daytime (10:00 to <15:00 h) did not differ significantly by sleep duration. Energy intake in the afternoon/evening (15:00 to <19:00 h) was significantly *lower* in the shorter‐sleeping groups in a linear fashion (*P* = 0.023), although the absolute differences were small; children sleeping <10 h a night consumed 319 kcal, while children in the longest‐sleeping group consumed on average 360 kcal, a difference of 41 kcal. Energy intake at night (19:00 to <6:00 h) was significantly higher in the shorter sleepers in a linear fashion (*P* < 0.001). Children in the shortest‐sleeping group (<10 h a night) consumed on average 166 kcal at night, while those in the longest (>13 h) consumed 46 kcal, an average difference of 120 kcal.

**Table 2:** Unadjusted dietary data by nighttime sleep duration (mean [standard deviation])

	<10 h	10–<11 h	11–<12 h	12–<13 h	≥13 h	*P* (linear trend)
Total energy intake (mean kcal per day)	1088.17 (195.35)	1046.83 (198.44)	1035.39 (188.24)	1025.83 (189.39)	1006.05 (202.39)	0.020
Total energy intake by eating occasion (mean kcal per day)						
Meals	691.65 (179.39)	674.20 (194.46)	715.99 (186.83)	707.48 (188.45)	703.12 (199.09)	0.473
Snacks	176.15 (137.50)	165.17 (115.51)	150.73 (101.38)	149.49 (110.97)	140.84 (98.20)	0.053
Drinks only	220.37 (154.09)	207.46 (120.26)	168.67 (111.02)	168.48 (119.39)	162.09 (131.42)	0.002
Total energy intake by time of day (mean kcal per day)						
Morning	253.43 (81.50)	275.32 (97.10)	285.65 (93.26)	277.15 (94.67)	260.06 (86.17)	0.698
Day	349.77 (116.85)	329.67 (94.14)	326.34 (96.71)	334.56 (95.52)	340.11 (120.76)	0.724
Afternoon/Evening	319.40 (129.86)	328.32 (116.99)	349.66 (114.06)	356.93 (114.78)	359.94 (119.80)	0.023
Night	165.55 (88.84)	113.52 (92.86)	73.74 (79.64)	57.19 (75.72)	45.94 (71.98)	<0.001
Total energy intake at night by eating occasion (mean kcal per night)						
Meals	25.44 (50.44)	9.59 (32.22)	6.77 (33.25)	4.68 (33.45)	5.04 (25.69)	0.001
Snacks	22.10 (37.13)	11.97 (43.64)	3.61 (17.28)	3.24 (22.39)	0.89 (7.41)	<0.001
Drinks only	118.02 (79.94)	91.96 (75.91)	63.37 (71.01)	49.28 (62.86)	40.00 (63.66)	<0.001

Over 90% of calories consumed during the nighttime period, across all sleep groups, were consumed before 12 midnight (range for 12:00–6:00 h was 3–10 kcal).

### Partitioning energy intake into meals, snacks and drinks

**Figure 1:**
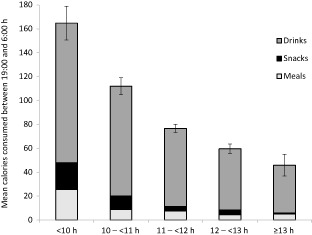
Energy intake at night (mean [standard error]) obtained separately from meals, snacks and drinks by nighttime sleep duration. Values are adjusted for age, sex, birth weight, gestational age, maternal education and daytime sleep (*P* for linear trend <0.001).

Across all drink occasions recorded, 76.8% were milk drinks and 98.6% of all calories in drink occasions came from milk drinks. Partitioning energy intake at night into energy obtained from meals, snacks and drinks showed that shorter sleep was associated with higher intake from all occasions (*P's* <0.001; Table 2). However, this amounted only to a 20‐kcal difference from shortest to longest sleepers from meal and snack occasions; the largest difference in nighttime calories were from drinks, which in this sample were almost all milk drinks (98.6%). On average, children in the shortest‐sleeping group consumed 118 kcal from drinks at night, while those in the longest consumed 40 kcal, with a linear pattern in between (adjusted values for calories at night are presented in Fig. 1).

Associations between sleep and energy intake (by time of day and eating occasion) were unchanged after adjusting for age, sex, birth weight, gestational age, maternal education, weight and daytime sleep (data not shown).

## Discussion

This study shows that shorter sleep in young children is associated with higher energy intake between 19:00 and 6:00 h and that the majority of additional intake comes from milk drinks consumed before midnight. This finding persisted after adjustment for multiple potential confounders. Nighttime consumption of milk drinks may be an important means through which shorter sleep contributes to adiposity and provides a potential target for intervention.

This is the first study to demonstrate that sleep is associated with temporal variations in energy intake in children. The findings are consistent with experimental data in adults and older children which have shown that consecutive nights of sleep restriction encourage the consumption of additional energy at night 6, 7, 9. Supporting the causal association, we were able to demonstrate a linear association between sleep duration and nighttime energy intake. Average nighttime intake went from 166 kcal in the shortest sleepers (<10 h) to 114, 74 and 57 to 46 kcal in the longest sleepers (>13 h). Our nighttime period extended to 6 in the morning, but almost all calories were consumed before midnight (over 90% across all sleep groups). This finding was observed before an association with weight had emerged. This has the advantage of precluding the possibility of confounding by weight status and suggests that a higher nighttime energy intake may be a mechanism through which sleep influences weight gain in early childhood, although longer‐term follow‐up is needed to demonstrate that these differences are sustained and significantly contribute to weight gain in early life. We also found that shorter sleepers consumed slightly less in the afternoon/evening period, but these differences were comparatively small and did not compensate for the higher energy intake at night.

We show that shorter‐sleeping children had both a later bedtime and an earlier wake time, but only consumed more than longer sleepers in the nighttime period. It may be that having more time to eat, particularly at night, contributes to excess energy intake among shorter sleepers. This could help explain why both short sleep and later sleep timing have been associated with weight gain in prospective studies of children 19. Indeed, our results show that among children sleeping <10 h a night, nighttime consumption alone accounted for 15.2% of daily energy intake. This is strikingly comparable with the findings on nighttime intake in adults exposed to sleep loss, which has ranged from 14% to 22% of daily energy intake 7. Among adults, the timing of food intake itself has been implicated in weight regulation, with later eating times being associated with higher BMI, poorer weight loss outcomes and a higher risk of metabolic abnormalities 20, 21, 22. The fact that the additional energy intake in shorter sleepers is consumed at night may therefore contribute to the development of adiposity, or its consequences, through more than a simple energy balance mechanism.

In this sample, the majority of nighttime calories were obtained from drinks, of which over 98% were from milk drinks. While we did not assess why parents were giving their children additional drinks at night, one possibility is that hunger levels may be endogenously higher in the evening 23, so parents could have been responding to perceived hunger. Alternatively, because provision of milk drinks is a common parental strategy to help young children initiate sleep 24, parents could have been providing milk to soothe their child before sleep or during the night. Although commonly used by parents to promote good sleep, feeding at night is usually associated with poorer sleep outcomes in young children 25, possibly because this practice prevents the child from developing the capacity to self‐soothe. Habits form when behaviours are repeated frequently in a stable context, with the context alone becoming sufficient to cue to the behaviour 26. Routinely providing milk before bed or as part of a bedtime routine may therefore result in nighttime consumption becoming habitual and dissociated from the child's physiological requirements.

Feeding before bed or during the night may be good practice in infants who are exclusively milk‐fed, but after weaning this should no longer be necessary 25. We show that providing milk drinks at night contributes to shorter sleepers consuming more energy, potentially contributing to later weight gain. If this practice also operates to dissociate feeding from hunger, and doesn't aid sleep, parents could be encouraged to use alternative methods to help initiate and consolidate nighttime sleep in young children.

### Strengths and limitations

This study benefited from detailed food diaries that recorded multiple days of dietary intake and included a timed entry for each eating occasion. This is the largest dietary data set of its kind for young children in the UK. The use of parent reports of sleep behaviour is a limitation, but this method is commonly used in large‐scale cohorts where objective measurements are not feasible 2. Encouragingly, the average nighttime sleep duration in this sample (11.6 h) is comparable with the published reference values for this age group 27. We categorized sleep duration into five groups at 1‐h intervals specifically to examine linear trends within the dietary data. Categorizing sleep in this way allows comparisons to be made with the sleep–weight literature, the majority of which has dichotomized sleep duration using a variety of cut points. However, when sleep duration was treated as a continuous variable, all associations with nighttime intake remained significant.

There was a slight time lag between the sleep (16 months) and dietary assessments (21 months). However, nighttime sleep duration is relatively stable at this age 27 and patterns of sleep behaviour tend to correlate over the first 3 years of life 28, so it is likely that sleep duration would be similar at the two times. Children with complete data had mothers who were more highly educated, had a lower BMI and were more likely to be from a white ethnic background. This could limit the generalizability of the findings, so replication in a more diverse sample is required.

A complex relationship exists between sleep, circadian rhythms and energy regulation 29 and we cannot exclude the possibility that energy intake at night disturbed subsequent sleep initiation or consolidation 30, particularly given that sleep disturbances and feeding problems can co‐exist in early life 31. However, it is encouraging that the findings were consistent with results from experimental studies of sleep restriction 6, 9. The results are also important in showing that temporal variation in energy intake exists under free‐living conditions and not only after experimentally induced sleep restriction. Identifying temporal patterning in energy intake among young children with limited feeding autonomy is also important given that associations between sleep and weight appear stronger in paediatric populations 1.

Although participants were from a cohort of twins, we included data from one twin selected at random from each pair. There is no reason to expect that any of the observed associations between sleep and eating behaviour would differ in singletons. It is also reassuring that the average daily caloric intake in this sample is comparable with the reference values for children of this age 32.

### Conclusion

We show that shorter‐sleeping children consume more energy than longer sleepers, that this is predominantly after 19:00 h, and the majority of the extra calories come from milk drinks. This could lead to simple guidance to parents to avoid excess energy intake in nighttime drinks for young children.

## Conflict of interest statement

No conflict of interest was declared.

## Author contributions

LM conceptualized the study, carried out the data coding and data analysis, drafted the initial manuscript and approved the final manuscript as submitted. JW conceptualized and designed the Gemini study, designed the data collection instruments, critically reviewed and revised the manuscript and approved the final manuscript as submitted. CHL supervised the data analysis, critically reviewed the manuscript and approved the final manuscript as submitted. LJ designed the dietary data collection instruments, coordinated the data collection, critically reviewed the manuscript and approved the final manuscript as submitted. CHMvJ supervised the data collection and data coding, critically reviewed the manuscript and approved the final manuscript as submitted. HS assisted with the data coding, critically reviewed the manuscript and approved the final manuscript as submitted. AF conceptualized the study, supervised the data analysis, critically reviewed and revised the manuscript and approved the final manuscript as submitted.

## Funding

Gemini was funded by a grant from Cancer Research UK (C1418/A7974). Coding of the diet diaries was supported by funding from DANONE Baby Nutrition (Nutricia Ltd). Laura McDonald is funded by a UK Medical Research Council PhD studentship.
